# Magnetless Optical Circulator Based on an Iron Garnet with Reduced Magnetization Saturation

**DOI:** 10.3390/molecules26154692

**Published:** 2021-08-03

**Authors:** Gianni Portela, Miguel Levy, Hugo E. Hernandez-Figueroa

**Affiliations:** 1 School of Electrical and Computer Engineering, University of Campinas, Campinas 13083-852, SP, Brazil; hugo@unicamp.br; 2 Physics Department, Michigan Technological University, Houghton, MI 49931, USA; mlevy@mtu.edu

**Keywords:** circulators, magneto-optical materials, optical communication systems

## Abstract

A three-port circulator for optical communication systems comprising a photonic crystal slab made of a magneto-optical material in which an magnetizing element is not required to keep its magnetic domains aligned is suggested for the first time. By maximizing the incorporation of europium to its molecular formula, the magneto-optical material can remain in the saturated magnetic state even in the absence of an external DC magnetic field. Two- and three-dimensional simulations of the device performed with full-wave electromagnetic solvers based on the finite element method demonstrate that, at the 1550 nm wavelength, the insertion loss, isolation, and reflection levels are equal to or better than −1 dB, −14 dB, and −20 dB, respectively. Since its operation does not require an electromagnet or a permanent magnet, the suggested circulator is much more compact, being able to reach footprints in the range of three orders of magnitude smaller, when compared to other circulator designs referred to in the literature and the presented results can be useful for the design of other nonreciprocal devices with reduced dimensions for optical communication systems.

## 1. Introduction

The operation of signal sources, like LASERs or LEDs, in an optical communication system is subject to instabilities caused by parasitic reflections arising from unmatched loads connected to the system. Nonreciprocal devices, like circulators and isolators, can be used to mitigate the effect of such reflections by absorbing or routing them to a matched load [1,2,3,4,5,6].

Conventional optical circulator designs referred to in the literature are based on the reciprocity breaking caused by the application of a static magnetic field to a material with magneto-optical (MO) properties. In order to provide an useful magneto-optical activity, the material must have its magnetic domains aligned by an external magnetizing element, like a permanent magnet or an electromagnet. Otherwise, the magnetic domains are randomly oriented and the MO effect is weakened.

These magnetizing elements are bulky and their utilization does not favor the design of circulators with a reduced footprint for optical communication systems with high integration density. For example, a three-port circulator based on a photonic crystal (PhC) structure is presented in [7]. In this case, three waveguides and one resonator are inserted in a photonic crystal made of a triangular lattice of holes etched in a bismuth iron garnet (BIG) film. The BIG thin film requires an external magnet to keep its magnetic domains saturated and the suggested design is feasible only at the 633 nm wavelength, with an estimated 213 GHz bandwidth for the 30 dB isolation level.

In [8], a four-port circulator comprising a ring resonator coupled to two waveguides for operation at the 1550 nm wavelength is suggested. The waveguides and the ring resonator are fabricated on a conventional silicon on insulator (SOI) wafer and a MO film made of a cerium-substituted yttrium iron garnet (Ce:YIG) is bonded on the silicon ring resonator. The operating bandwidth of the device around the 1550 nm wavelength calculated from computational simulations is about 5 GHz (taking into consideration the 9 dB isolation level). The external DC magnetic field required for the magnetization of the Ce:YIG film is provided by an electromagnet based on a gold microstrip coil. Since the electromagnet demands an electric current for its operation, additional issues beyond the size of the magnetizing element, such as current supply and Joule heating, can hinder the utilization of the circulator in integrated optical circuits.

Another optical circulator design is suggested in [9]. The presented design is based on a PhC made of a triangular lattice of holes drilled in a BIG film in which three waveguides and a multi-ring resonator are inserted. A moderate static external magnetic field (<0.5 T) is required for the BIG saturation and the off-diagonal element *g* of the BIG permittivity tensor in the order of 0.1 is not feasible at the 1550 nm wavelength, but only at shorter ones (around 620 nm). Numerical calculations of the suggested circulator show that its operating bandwidth around the 620 nm wavelength is about 170 GHz for the 20 dB isolation level.

In order to overcome such limitations of the circulator designs reported in the literature, we suggest in this paper a compact PhC-based three-port circulator that can operate at the 1550 nm wavelength and, most importantly, whose operation does not require an external DC magnetic field. The magnetless operation is possible because the MO material used in the design, a commercially available bismuth-substituted iron garnet with the incorporation of europium and molecular formula ${Bi}_{X}{\left(Eu_{Z}Ho_{1–Z}\right)}_{3-X}Fe_{5-Y}Ga_{Y}O_{12}$ [10,11], does not require external magnetic fields to keep its saturated magnetic state.

Since it does not require external magnetizing elements, the presented circulator is more compact and its utilization in optical circuits with high integration density operating at the 1550 nm wavelength is more feasible in comparison to other designs already reported. Specifically in comparison to the design presented in [8], the footprint of the proposed circulator is three orders of magnitude smaller.

The performance characteristics of the suggested circulator design (center frequency, S-parameters, and operating bandwidth) have been obtained from two- and three-dimensional simulations of the circulator performed with the full-wave electromagnetic solvers COMSOL Multiphysics and CST Studio Suite, both based on the Finite Element Method (FEM), and the numerical results evidence the feasibility of the proposed circulator design.

Moreover, we briefly discuss the utilization of the temporal coupled-mode theory (TCMT) method for the analysis of the proposed circulator and show that the results derived from TCMT equations are in good agreement with those obtained from the computational simulations.

## 2. Circulator Design

**Figure 1:**
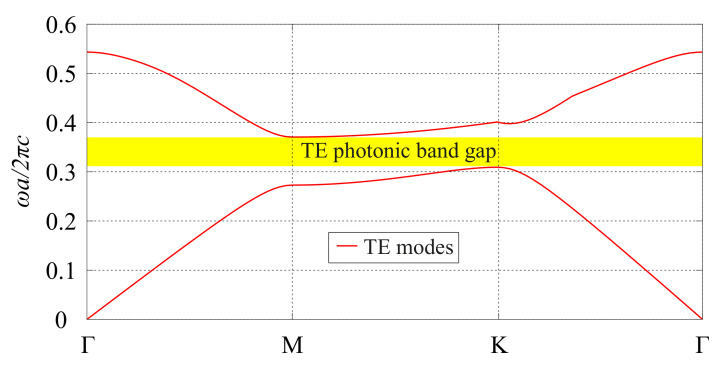
Dispersion relation of the PhC structure for TE modes (only the first and second bands are shown).

A photonic crystal consisting of a triangular lattice of holes with lattice constant *a* drilled in a slab of the MO material with thickness h=a has been considered in the design of the optical circulator. The radius of the air holes is r=0.3a and, for operation at the 1550 nm wavelength, we have a=505 nm. The dispersion relation of this periodic structure for TE polarization has been calculated with the MIT Photonic Bands (MPB) package and it is presented in Figure 1. One can see that there is a photonic band gap (PBG) between the first and second TE bands (corresponding to the normalized frequency range ωa/2πc = 0.3092–0.3706). We have considered the conventional notation in the PhC literature for TE modes, in which the electric field is confined to the periodicity plane of the PhC (xy-plane in this paper) [12].

**Figure 2:**
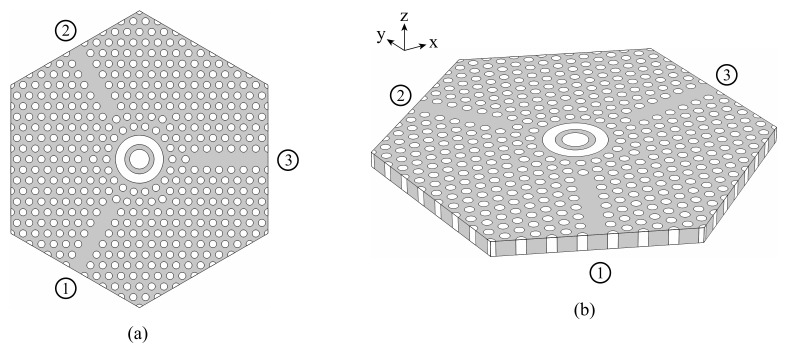
(**a**) Top view and (**b**) perspective view of the circulator.

The final design, including the waveguides and the resonator, is presented in Figure 2. The gray regions are made of the MO material, while the white ones are filled with air.

**Figure 3:**
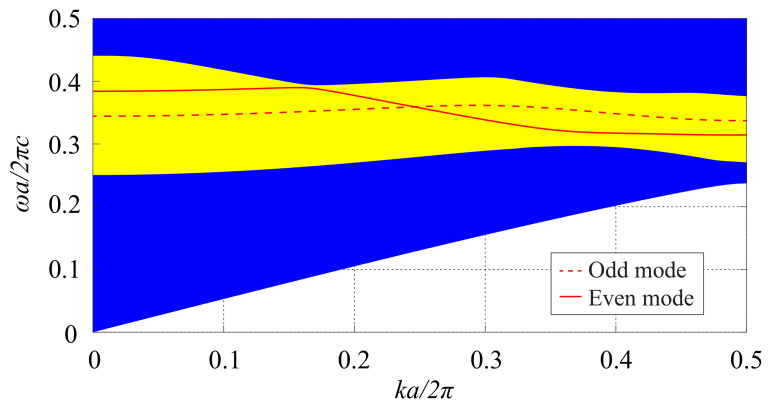
Dispersion diagram of the PhC W1 waveguides.

Each of the three waveguides is created by removing a single row of air holes from the periodic structure (W1 waveguides) and is frontally coupled to the center resonator. The dispersion diagram of the waveguides, calculated with the MPB package, is shown in Figure 3. As one can see from the dispersion diagram of the waveguides, they support both odd and even modes in the PBG range. In order to obtain a single-mode operation, we have designed the resonator so that the resonant frequencies of its counter-rotating dipole modes lie in the frequency range in which only the even mode of the waveguide can propagate.

Regarding the resonator, we have adapted for our purposes the resonator structure suggested in [9]. It consists of a center enlarged hole surrounded by concentric rings (either true rings or mimicked by holes arranged on a circle). The resonator supports counter-rotating dipole modes with resonant frequencies $\omega_{+}=1.215053\times 10^{15}$ rad/s and $\omega_{-}=1.215082\times 10^{15}$ rad/s. More details regarding the resonator geometry are given in Appendix A.

**Figure 4:**
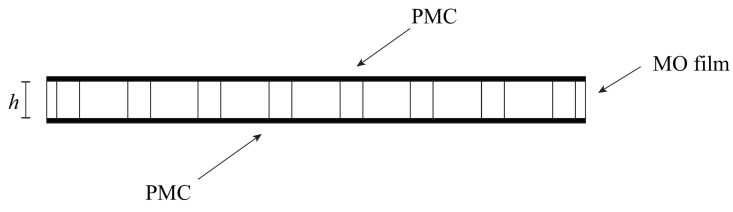
Cross-section of the circulator.

The resonant modes are mainly confined to the center enlarged hole of the resonator and, as a consequence, they are very leaky in the vertical direction (along z-axis) if the MO film is simply surrounded by air (the refractive index contrast in this case is very low). In order to provide the vertical confinement of the resonant modes and avoid energy leakage, we have considered in our 3D simulations that perfect magnetic conductor (PMC) layers cover the top and bottom boundaries of the MO film, as schematically shown in Figure 4.

### 2.1. Magneto-Optical Material Properties

We have considered a bismuth-substituted iron garnet with the incorporation of europium and molecular formula ${Bi}_{X}X{\left(Eu_{Z}Ho_{1–Z}\right)}_{3-X}Fe_{5-Y}Ga_{Y}O_{12}$ in the circulator design. The main difference between this material and other MO materials commonly used in the design of optical nonreciprocal devices, like YIG (Yttrium Iron Garnet), BIG (Bismuth Iron Garnet), Ce:YIG (Cerium-substituted Yttrium Iron Garnet) or Bi:YIG (Bismuth-substituted Yttrium Iron Garnet), is that the former does not require static external magnetic fields to keep its magnetic domains saturated, whereas the others do. 

This is possible because the incorporation of europium (Eu) to the molecular formula of the MO film is maximized, with consequent reduction in the magnetization saturation of the MO material (between 10 G and 60 G) without the creation of compensation point [10,11,13]. This material belongs to the crystallographic space group Ia3d and the *X*, *Y*, and *Z* parameters in its formula unit are the bismuth concentration, gallium concentration, and the europium fraction of rare earth in the film, respectively. We have considered in our numerical calculations a MO film with Z=0.45, X=1.22, and Y=0.956, that is, with a composition given by the formula unit ${Bi}_{X}1.22{\left(Eu_{0.45}Ho_{0.55}\right)}_{1.78}Fe_{4.044}Ga_{0.956}O_{12}$ with eight formula units per crystal unit cell.

The MO material has, at the 1550 nm wavelength, a refractive index (*n*) and specific Faraday rotation ($\theta_{F}$) equal to 2.3 and 930 deg/cm, respectively [13,14]. Furthermore, the magnetic permeability of the MO material is $\mu =\mu_{0}$ and its electric permittivity tensor is defined as follows:

(1) $$\left[\epsilon \right]=\epsilon_{0}\left(\;\;\begin{array}{ccc} \;\epsilon_{r}\; & -ig\; & 0\;\\ \;ig\; & \epsilon_{r}\; & 0\;\\ \;0\; & 0\; & \epsilon_{r}\; \end{array}\;\;\right),$$

where $\epsilon_{r}=n^{2}$ and the off-diagonal element g≈0.002 (gyrotropy) is proportional to $\theta_{F}$.

The insertion loss of the material is less than or equal to 0.05 dB for 45 degrees Faraday rotation at the 1550 nm wavelength [14]. Considering that the thickness for 45 degrees Faraday rotation is about 480 μm, the loss per micron of the material is very small (less than 0.0001 dB). For this reason, we have neglected the effect of material losses in our computational simulations.

### 2.2. Implementation of the Perfect Magnetic Conductor Layers

It is well known that metals (like copper) can be regarded, in an approximation, as perfect electric conductor (PEC) materials in the microwave frequency range. However, there is not a naturally occurring material that can be regarded as a PMC, that is, a material in which the tangential magnetic field is zero at its surface.

In order to satisfy the requirement of our device for top and bottom PMC layers, the main alternative is the design of metamaterials that can emulate the properties of a PMC surface. For example, the authors of [15] present an all-dielectric metasurface based on a subwavelength 2D array of dielectric resonators made of tellurium (Te) that shows a PMC behavior in the optical frequency range.

On the other hand, high impedance surfaces (HIS) can be also employed for the practical implementation of a PMC. For instance, the authors of [16] suggest a new method for the miniaturization of microwave resonators based on the combination of PEC and PMC layers, with the latter being realized by the utilization of a HIS structure comprising a periodic array of metallic patches on a dielectric substrate confined by the metallic walls of a cavity.

In addition, the authors of [17] suggest the utilization of epsilon-near-zero (ENZ) media with engineered dielectric defects that shows the same scattering properties of an ideal PMC. In this case, the PMC-like behavior of the media can be mimicked from the microwave to infrared bands and it is related to the excitation of magnetic resonances in the defects and to the localization of the nodes of standing waves arising from the resonances on the boundary of the defects.

The practical implementation of a PMC surface and its integration to the suggested circulator, as schematically shown in Figure 4, is beyond the scope of this paper, since our main objective is to present the design of an optical circulator with magnetless operation. We intend to show in a future paper how to address this question by designing a metamaterial for the emulation of the PMC behavior, as suggested in [15,16,17], and incorporating it to the presented circulator structure.

## 3. Scattering Matrix Analysis of the Device

**Figure 5:**
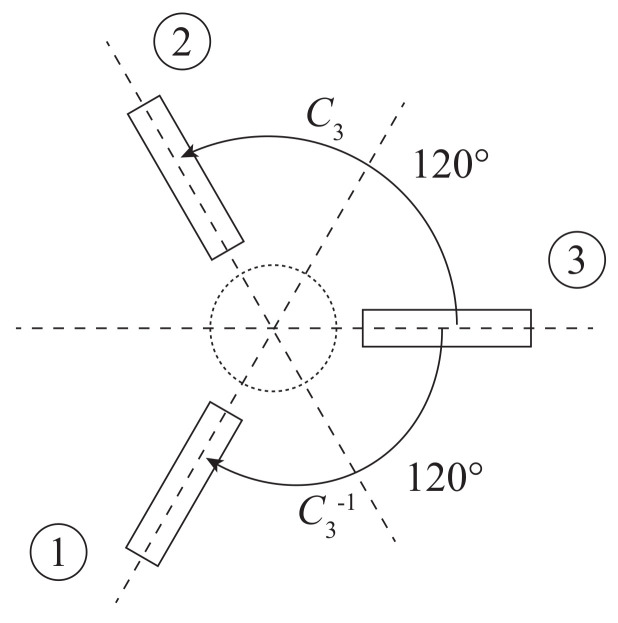
Symmetry elements $C_{3}$ and $C_{3}^{-1}$.

The proposed circulator has a three-fold rotational symmetry, that is, the unitary symmetry elements $C_{3}$ (counterclockwise rotation by 2π/3) and $C_{3}^{-1}$ (clockwise rotation by 2π/3) are contained in the symmetry group of the device (see Figure 5). The structure of the scattering matrix *S* of the circulator can be derived from the commutation relations $R_{C_{3}}S=SR_{C_{3}}$ or $R_{C_{3}^{-1}}S=SR_{C_{3}^{-1}}$, where $R_{C_{3}}$ and $R_{C_{3}^{-1}}$ are the representation matrices of the elements $C_{3}$ and $C_{3}^{-1}$, respectively [18].

It is sufficient to consider only one of these two elements for the symmetry analysis of the circulator scattering matrix *S*, since the relations between the S-matrix elements obtained from the commutation relations are the same for both symmetry elements [18]. Therefore, we will consider only the element $C_{3}$ in our analysis from now on.

The representation matrix $R_{C_{3}}$ of the element $C_{3}$ can be defined as follows:

(2) $$R_{C_{3}}=\left(\;\begin{array}{ccc} 0 & 0 & 1\\ 1 & 0 & 0\\ 0 & 1 & 0 \end{array}\;\;\right).$$

By taking into consideration Equation (2) and the commutation relation $R_{C_{3}}S=SR_{C_{3}}$, one can derive the following frequency independent relations between the S-matrix elements: $S_{11}=S_{22}=S_{33}$, $S_{21}=S_{32}=S_{13}$, and $S_{31}=S_{12}=S_{23}$. They allow us to define the following scattering matrix of the circulator:

(3) $$S=\left(\;\begin{array}{ccc} S_{11} & S_{31} & S_{21}\\ S_{21} & S_{11} & S_{31}\\ S_{31} & S_{21} & S_{11} \end{array}\;\;\;\right).$$

One can see from Equation (3) that the scattering matrix of the circulator has only three independent elements. Therefore, there is no need to apply the input signal to each of the three ports of the device in order to obtain its full scattering matrix, but only to a single port.

The three independent entries of the circulator S-matrix can be experimentally measured with a vector network analyzer or numerically calculated with full-wave electromagnetic solvers. It is also possible to derive formulas for their calculation from the analytical TCMT method [12,19,20].

Analytical models based on TCMT are very useful for the analysis of resonant devices with weak decay rates. They are based on the following general assumptions: weak coupling, time-invariance of the design (material properties and geometry do not depend on time), linearity, conservation of energy, and time-reversal invariance [12]. The most important assumption and the only one that cannot be relaxed in order to obtain a quantitatively accurate model is weak coupling [12]. Therefore, the TCMT method can be used even for the description of nonreciprocal devices (like circulators and isolators), where time-reversal invariance is not conserved.

A general TCMT based model for a W-circulator with low symmetry described by a single antiunitary element is presented in [21]. The suggested model can be easily adapted for our case with three-fold rotational symmetry by considering that $\varphi =\varphi_{23}-\varphi_{12}=0$, where $\varphi_{23}$ and $\varphi_{12}$ are the phases of the S-matrix entries $S_{23}$ and $S_{12}$, respectively. Therefore, the TCMT equations for the calculation of the circulator S-matrix are [21]:

(4) $$S_{11}=S_{22}=S_{33}=-1+\frac{2}{3}\left(\frac{1}{1+i\left(\omega -\omega_{+}\right)/\gamma_{+}}+\frac{1}{1+i\left(\omega -\omega_{-}\right)/\gamma_{-}}\right),$$

(5) $$S_{21}=S_{32}=S_{13}=\frac{2}{3}\left(\frac{e^{i2\pi /3}}{1+i\left(\omega -\omega_{+}\right)/\gamma_{+}}+\frac{e^{-i2\pi /3}}{1+i\left(\omega -\omega_{-}\right)/\gamma_{-}}\right),$$

(6) $$S_{31}=S_{12}=S_{23}=\frac{2}{3}\left(\frac{e^{-i2\pi /3}}{1+i\left(\omega -\omega_{+}\right)/\gamma_{+}}+\frac{e^{i2\pi /3}}{1+i\left(\omega -\omega_{-}\right)/\gamma_{-}}\right),$$

where $\omega_{+}$, $\gamma_{+}$, $\omega_{-}$, and $\gamma_{-}$ are the resonant frequency of the $a_{+}$ mode, the decay rate of the $a_{+}$ mode, the resonant frequency of the $a_{-}$ mode, and the decay rate of the $a_{-}$ mode, respectively. In our case, $a_{+}$ and $a_{-}$ are counter-rotating dipole modes.

We will show in Section 4 that there is a good agreement between the results provided by Equations (4)–(6) and those obtained from the computational simulations of the device. It is worth noting that these equations are very general and can be used to describe similar Y-shaped circulators with three-fold rotational symmetry based on different technologies (e.g., PhC, microstrip, stripline, etc.).

An interesting result that can be directly derived from Equations (4)–(6) is the formula for the fractional bandwidth of the circulator [21]. It can be shown that:

(7) $$\frac{\Delta f}{f_{0}}=k\frac{|\omega_{+}-\omega_{-}|}{\omega_{0}}$$

where Δf is the absolute bandwidth, $f_{0}$ is the center frequency, $\omega_{0}=2\pi f_{0}$, and *k* is a constant whose value depends only on the reference isolation level (usually −10 dB or −15 dB).

Therefore, the circulator bandwidth is proportional to the frequency splitting $|\omega_{+}-\omega_{-}|$ of the counter-rotating dipole modes excited in the resonator. The main alternative for increasing the frequency splitting and, as a consequence, the circulator bandwidth is the utilization of MO materials with high Faraday rotation, since the frequency splitting is proportional to the off-diagonal element *g* (gyrotropy) of the permittivity tensor of the MO material, which in turn is proportional to the specific Faraday rotation parameter $\theta_{F}$.

The value of $\theta_{F}$ of some MO materials at the 1550 nm wavelength can be up to ten times greater than that of the MO material that we used in our design [8,22,23]. However, such materials require an external magnetizing element to keep its saturated magnetic state. Consequently, the magnetless operation of our circulator design comes at the expense of a lower operating bandwidth.

## 4. Results

We have performed two- and three-dimensional simulations with the full-wave electromagnetic solvers COMSOL Multiphysics and CST Studio Suite in order to demonstrate the feasibility of the magnetless optical circulator. By taking into consideration the symmetry aspects described in Section 3, we have considered that the input signal is applied only to port 1 in order to calculate the full frequency dependent S-matrix of the device. In addition, we have calculated the S-parameters of the device with Equations (4)–(6) and compared the analytical results with those obtained from computational simulations.

### 4.1. Two-Dimensional Simulations with COMSOL Multiphysics

**Figure 6:**
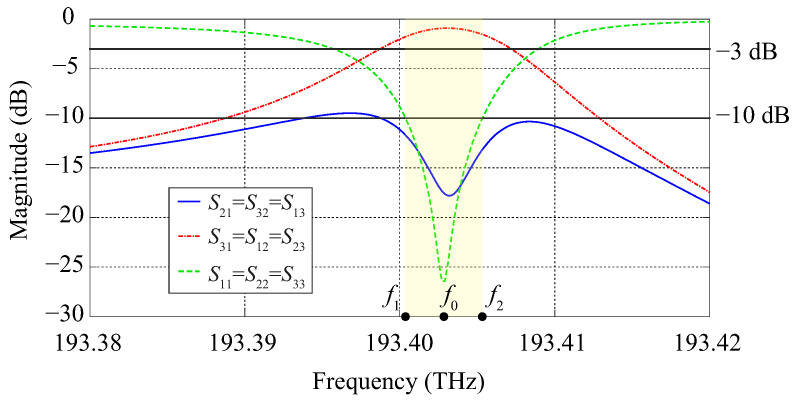
Frequency response obtained from 2D simulations performed with COMSOL Multiphysics.

**Figure 7:**
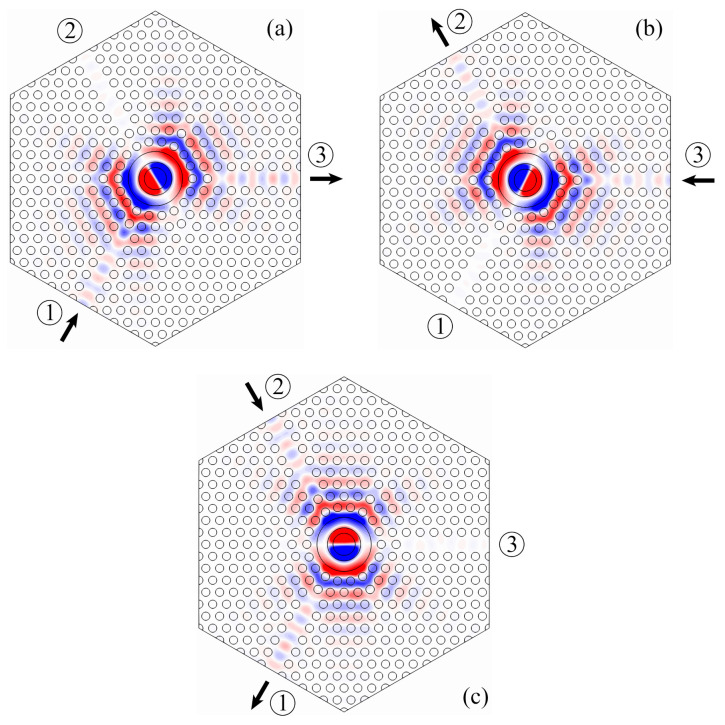
$H_{z}$ distribution at the center frequency $f_{0}$ obtained from 2D simulations performed with COMSOL Multiphysics for excitation at (**a**) port 1, (**b**) port 3, and (**c**) port 2.

Two-dimensional simulations of the device have been performed with the software COMSOL Multiphysics. In this case, the photonic crystal structure is considered infinite in the z-direction, for the sake of simplicity. The calculated frequency response is shown in Figure 6 and the field profile of the $H_{z}$ component in the circulator at the center frequency is shown in Figure 7.

The calculated insertion loss, isolation, and reflection levels at the center frequency $f_{0}=193.4028$ THz (corresponding to $\lambda_{0}=1550.094$ nm) are −1 dB, −18 dB, and −26 dB, respectively. The lower and upper frequency limits of the circulator band (highlighted in yellow in Figure 6), defined at the levels −3 dB of the $S_{31}$ curve and −10 dB of the $S_{21}$ and $S_{11}$ curves, are $f_{1}=193.4003$ THz and $f_{2}=193.4053$ THz, respectively. The operating bandwidth Δf, defined as $\Delta f=f_{2}-f_{1}$, is 5 GHz.

It is possible to verify from Figure 7 that the $H_{z}$ field distribution of the dipole mode is about the same in the input and output waveguides, while the nodal plane of the dipole is always aligned with the isolated waveguide. This specific alignment of the dipole mode is crucial for the proper functioning of the circulator and it can be adjusted by tuning the resonator geometry and the resonator–waveguide coupling.

### 4.2. Three-Dimensional Simulations with COMSOL Multiphysics

**Figure 8:**
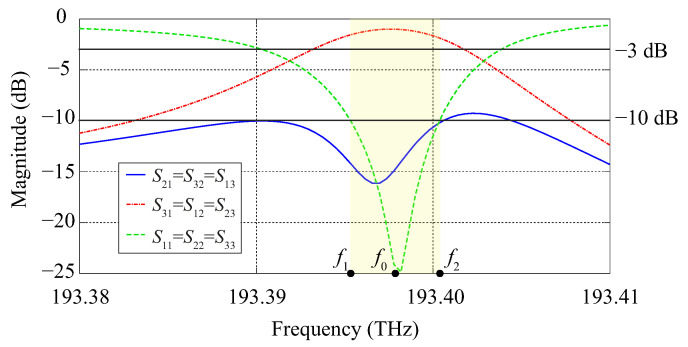
Frequency response obtained from 3D simulations performed with COMSOL Multiphysics.

**Figure 9:**
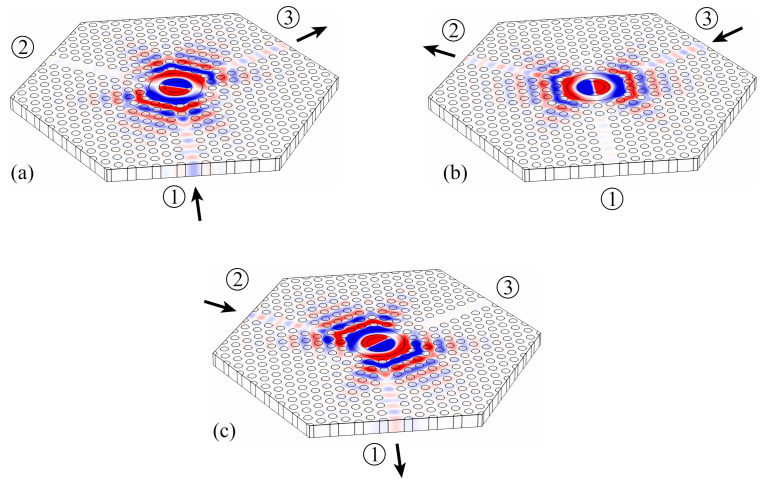
$H_{z}$ distribution at the center frequency $f_{0}$ obtained from 3D simulations performed with COMSOL Multiphysics for excitation at (**a**) port 1, (**b**) port 3, and (**c**) port 2.

We have also performed three-dimensional computational simulations of the circulator with the software COMSOL Multiphysics. The photonic crystal structure in the xy-plane is the same considered in Section 4.1, with the only difference that we are now considering a more realistic photonic crystal slab with finite thickness h=a bounded by PMC layers. The S-parameter curves and the $H_{z}$ field profile at the center frequency obtained from the 3D calculations are presented in Figure 8 and Figure 9, respectively.

At the center frequency $f_{0}=193.3978$ THz (equivalently, $\lambda_{0}=1550.134$ nm), the insertion loss is −1 dB, while the isolation and reflection levels are −15 dB and −24 dB, respectively. The frequency band of the circulator (highlighted in yellow in Figure 8) is defined by the lower and upper limits $f_{1}=193.3953$ THz and $f_{2}=193.4003$ THz, respectively, with a operating bandwidth $\Delta f=f_{2}-f_{1}=5$ GHz.

One can see from Figure 8 that there is a slight dislocation of the $S_{21}$ peak in comparison to the $S_{31}$ and $S_{11}$ peaks in our 3D calculations performed with COMSOL Multiphysics, which in turn is not noticeable in our 2D simulations. Nonetheless, one can see from Figure 6 and Figure 8 that there is a good agreement between the results provided by the 2D and 3D simulations performed with COMSOL Multiphysics.

### 4.3. Three-Dimensional Simulations with CST Studio Suite

In order to confirm the feasibility of the circulator, three-dimensional simulations of the circulator have also been performed with the software CST Studio Suite. The photonic crystal slab structure considered in this case is the same when compared to the one presented in Section 4.2. However, in the numerical calculations with CST Studio Suite, we have verified that it is better to attach a strip waveguide to each of the PhC waveguides and excite the strips in order to lower the reflection levels of the structure. This is not required in the simulations with COMSOL Multiphysics.

**Figure 10:**
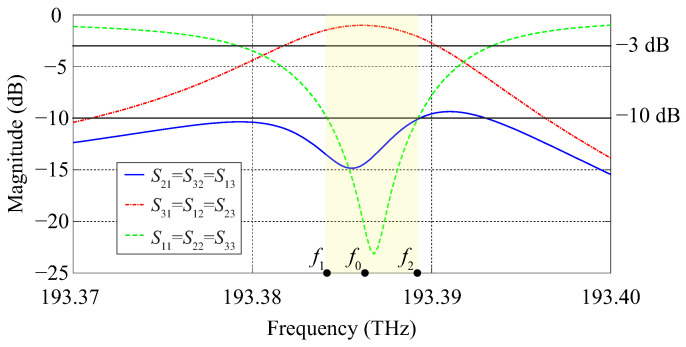
Frequency response obtained from 3D simulations performed with CST Studio Suite.

**Figure 11:**
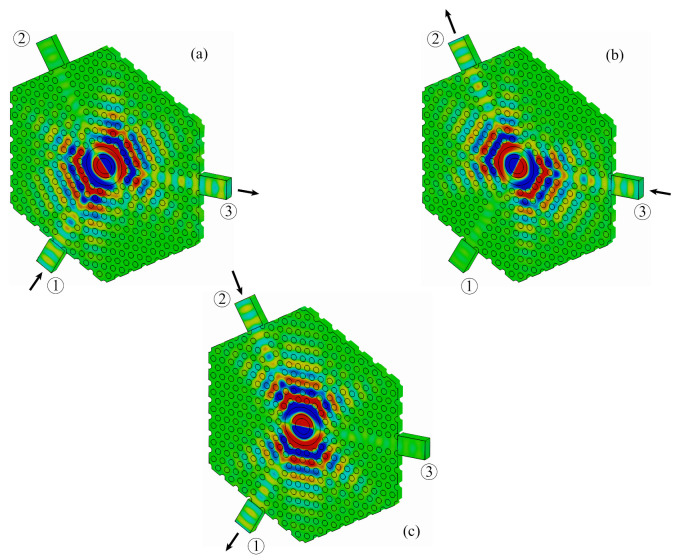
$H_{z}$ distribution at the center frequency $f_{0}$ obtained from 3D simulations performed with CST Studio Suite for excitation at (**a**) port 1, (**b**) port 3, and (**c**) port 2.

The frequency response and the field profile of the $H_{z}$ component at the center frequency calculated with CST Studio Suite are shown in Figure 10 and Figure 11, respectively. We have considered, in our calculations with CST Studio Suite, 1-μm wide strip waveguides with the same thickness of the PhC slab.

The calculated values for insertion loss, isolation, and reflection levels are −1 dB, −14 dB, and −20 dB, respectively, at the center frequency $f_{0}=193.3862$ THz (corresponding to $\lambda_{0}=1550.227$ nm). The frequency band of the circulator is highlighted in yellow in Figure 10 and its upper and lower limits are $f_{1}=193.3842$ THz and $f_{2}=193.3892$ THz, with an operating bandwidth $\Delta f=f_{2}-f_{1}=5$ GHz.

By comparing Figure 8 and Figure 10, one can see that the three-dimensional computational simulations of the magnetless optical circulator performed with the full-wave electromagnetic solvers COMSOL Multiphysics and CST Studio Suite produce similar results, with both demonstrating the feasibility of the device for operation at the 1550 nm wavelength.

### 4.4. Analytical Results Obtained from TCMT Equations

**Figure 12:**
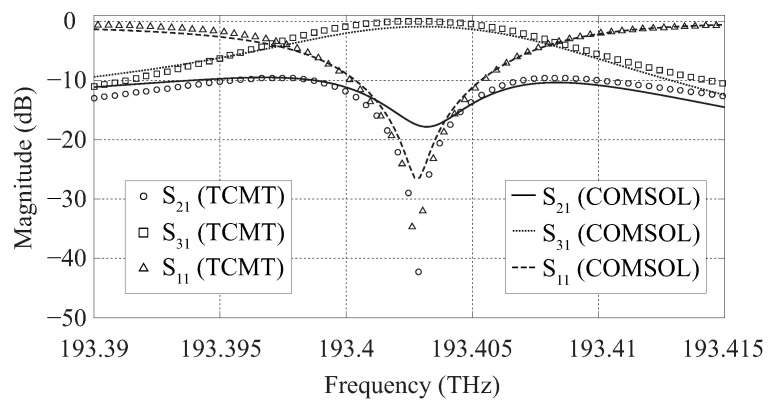
S-parameters of the circulator obtained from TCMT equations and 2D simulations with COMSOL Multiphysics.

We have also calculated the S-parameters of the circulator with Equations (4)–(6) in order to show that the device performance can be analytically predicted from a set of simple equations derived from a TCMT-based approach. Figure 12 presents a comparison between the analytical results obtained from the TCMT equations and those obtained from the 2D calculations performed with COMSOL Multiphysics.

One can see from Figure 12 that there is a good agreement between the analytical results obtained from Equations (4)–(6) and those obtained from the two-dimensional simulations performed with COMSOL Multiphysics. In our TCMT calculations we have considered that $\gamma_{+}=\gamma_{-}=3\times 10^{10}$ rad/s and that the condition $\omega_{0}=\omega_{-}-\gamma_{-}/\sqrt{3}=\omega_{+}+\gamma_{+}/\sqrt{3}$ is satisfied [21].

## 5. Discussion

The results obtained from all the computational simulations performed with the programs COMSOL Multiphysics and CST Studio Suite are summarized in Table 1, for better comparison. The two-dimensional simulation performed with COMSOL Multiphysics is referred to as “2D-COMSOL”, while the three-dimensional simulations performed with COMSOL Multiphysics and CST Studio Suite are referred to as “3D-COMSOL” and “3D-CST”, respectively.

One can see from Table 1 that there is a good agreement between the results provided by all the computational simulations performed with the softwares COMSOL Multiphysics and CST Studio Suite. We believe that the slight differences observed in Table 1 are related to peculiarities of the two solvers concerning the implementation of the FEM method.

Moreover the TCMT method can be very useful for the analysis of the suggested circulator. The formulas for the calculation of the circulator S-matrix and its fractional bandwidth obtained from the TCMT method allow one to analytically predict the performance of the device. The results derived from these formulas are in good agreement with those obtained from our computational simulations, as shown in Figure 12. More details concerning the TCMT method and its application to the analysis of nonreciprocal devices are given in [21].

## 6. Conclusions

The numerical results obtained from the computational simulations of the device demonstrate, for the first time, that the operation of a magnetless optical circulator at the 1550 nm wavelength is feasible. The suggested circulator does not require external magnetizing elements because it is made of an MO material that can keep its saturated magnetic state even without bias magnets.

Since it does not require electromagnets or permanent magnets for its proper functioning, the proposed circulator is much more compact when compared to conventional circulator designs that require bias magnets already reported in the literature. The design of optical communication systems with high integration density can benefit from the proposed circulator with reduced dimensions.

More specifically, the footprint of the suggested circulator is about 18.5 μm${}^{2}$, while the footprint of the circulator presented in [8], in which an electromagnet is required, is about 3203.5 μm${}^{2}$. Therefore, our design presents a footprint reduction by three orders of magnitude when compared to the design shown in the reference.

## Figures and Tables

**Table 1:** Results obtained from the performed computational simulations.

Simulation	$f_{0}$ (THz)	$\lambda_{0}$ (nm)	$S_{31}$ (dB)	$S_{21}$ (dB)	$S_{11}$ (dB)	Δf (GHz)
2D-COMSOL	193.4028	1550.094	−1	−18	−26	5
3D-COMSOL	193.3978	1550.134	−1	−15	−24	5
3D-CST	193.3862	1550.227	−1	−14	−20	5

**Figure A1:**
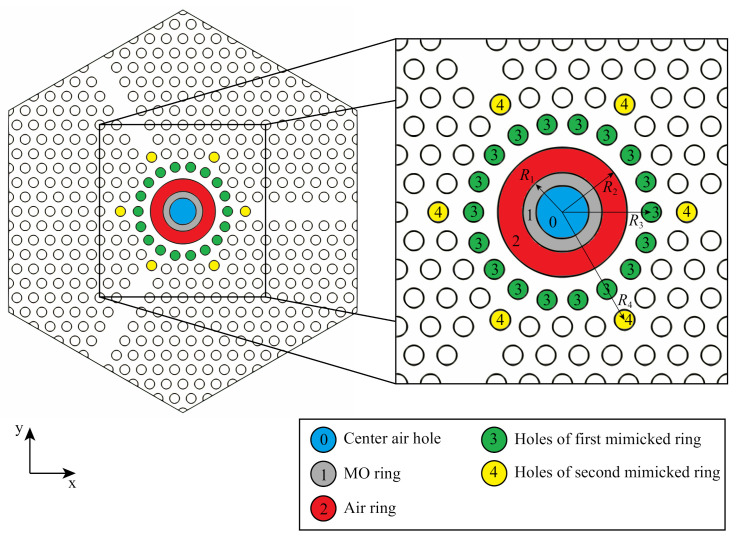
Top view of the circulator (resonator geometry is highlighted in the inset).

## Data Availability

Data is contained within the article.

## Appendix A. Details of Resonator Geometry

A top view of the circulator is presented in Figure A1. The white circles represent the PhC air holes drilled in the MO material. The radius of the air holes is 0.3a (a=505 nm for operation at the 1550 nm wavelength).

The resonator is located in the center part of the circulator (numbered air/MO regions with different colors) and it is highlighted in the inset of Figure A1. The radius of the blue center air hole (No. 0) is 0.805a. The outer radius of the gray MO ring (No. 1) is $R_{1}=1.203a$. The outer radius of the red air ring (No. 2) is $R_{2}=1.963a$. The radius of the 18 green air holes (No. 3) is 0.3a and these holes mimic a ring with radius $R_{3}=2.694a$. Finally, the radius of the six yellow air holes (No. 4) is 0.31a and these holes mimic a ring with radius $R_{4}=3.765a$.
